# Under What Conditions May Ipicoectomy Be Successful

**Published:** 1922-06

**Authors:** C. J. Lyons


### UNDER WHAT CONDITIONS MAY IPICOECTOMY BE SUCCESSFUL

BY C. J. LYONS, D.D.S.

It must be perfectly clear now that pathologic conditions of the peridental membrane, including that overlying the apices of the roots of teeth may be contributing factors to and frequently are the primary causes of general systemic disturbances.

With the present methods of root canal therapy, few of these morbid conditions existing at the apices of pulpless teeth which we call granulomata can be satisfactorily eliminated. Can they be eradicated satisfactorily by surgical procedure? This is a question that has been discussed pro and con by the writers on dental topics for many years.

A few years ago, when the "wholesale" extraction of teeth as a means of controlling general systemic conditions was at its height, my co-workers and myself at the University of Michigan determined to exert every effort to save the single rooted teeth. To this end we revived the old operation recently known as apicoectomy, which was begun back in 1884 by Farrar. We laid down four cardinal principles which must be carried out in every single case.

### FUNDAMENTAL PRINCIPLES IN ROOT AMPUTATION

First. A correct diagnosis of every case.

Second. Recognition and maintenance of surgical asepsis in the operative procedure.

Third. Elimination of the infection in the canals and tubuli and curettage of the involved area.

Fourth. Sealing the exposed root end after making the resection.

After four years of observation of cases operated upon the above principles, our results have satisfied us that these cases can be successfully treated by apicoectomy, if these principles are followed.

The first principle upon which we must govern our operative procedure is not an easy one of fulfillment. No hard and fast lines can be drawn as to when apicoectomy is indicated and when the extraction of the tooth should be the operation of choice. A correct diagnosis of the case is not a simple matter. Perhaps the most vital point to be considered in determining the character of the operation, that is, whether it is to be extraction of the tooth or apicoectomy, is the physical condition of the patient. The lowering of the vitality through disease, which leads to a state of constitutional dyscrasia, will so influence the process of repair that success would be uncertain in such cases.

The question of sterilizing and properly filling the canals in multi-rooted teeth for the elimination of infection is at this time a rather doubtful procedure, and in making the diagnosis, this operation should be limited to those teeth in which there is no question as to the operator's ability to sterilize and fill the root canals. In no case should this operation be attempted when the tissues are diseased beyond the apical third of the root of the tooth.

### SURGICAL ASEPSIS

The second principle to be carried out is the one of surgical asepsis in the operative procedure. The same general precautions should be exercised in carrying out this principle that are necessary in any other operation on human tissues in which the bone is involved.

### ROOT CANAL STERILIZATION

That part of the third principle which invloves the preparation of the root canal for the operation, is carried out by following the principles laid down by Howe in his paper on "A Method of Sterilizing and at the Same Time Impregnating with a Metal, Affected Dentinal Tissues," *Dental Cosmos,* September 1917, and by Rickert, "Advantages and Disadvantages of Reduced Silver in Dental Therapeutics," *Jr. N. D. A.*, October 1919.

That part of the third principle relating to the curettage of the involved area is carried out by mechanically removing all of the gross sepsis and disintegrated tissues from the crypt and polishing the margin of the crypt and the resected root end.

### TREATMENT OF ROOT END

The polishing away of all rough surfaces of alveolar process and root end is essential. No surfacce should be left that might set up an irritation during the process of repair. One of the causes of failure in the operation has been in leaving a rough root end. For years we have felt that one of the most prolific causes of failure has been that the end of the root with its open tubuli has been left exposed. Many schemes have been resorted to in the past to procure a protection for the exposed root end. None, however, seem to have met the requirements of completely sealing the exposed surfaces.

The exposed root must be sealed * with rapid reducing silver made up as follows: To a 10 per cent. or 15 per cent. solution of AgNo3, enough KOH or NaOH is added to convert all of the silver to the oxide. The oxide is carefully washed with distilled water and placed in an amber colored bottle for future use. This preparation at this stage is so unstable that strong light will be sufficient to throw down the silver.

When the rapid reducing silver is desired, the oxide is carried with a glass rod into a drop or two of pure NH3OH until the fumes of ammonia are no longer noticeable. The reaction results in the formation of silver ammonium oxide. This is then applied to the root end which has been previously dried. It is left on the end of the root from three to five minutes, after which a small pledget of cotton saturated with eugenol or formalin is applied and left on one minute. The excess is removed and the resected end of the root is burnished with a warm burnisher. The treatment may be repeated until a dense black layer of silver is deposited over the root end. All unused silver ammonium oxide should be discarded immediately after use because of the possibility of the formation of explosive fulminants of silver.

After the process of reducing silver over the exposed root end is completed, a blood clot is encouraged and the wound may be closed. The success or failure of this operation does not depend upon a certain exact technic of operative procedure, but does depend upon the carrying out in detail of the four principles as previously suggested.

Just what takes place in the crypt following the operation is a question that has been discussed somewhat extensively. Some men assert that the crypt is filled in with sclerotic bone, that is, a dense, hard, non-vascular bone. Our observations have not been in accord with these findings. We feel that the crypt becomes filled with bone quite identical to that of the normal area surrounding it.

In fact, if the principles above stated are carried out, we have left a condition that does not vary greatly from any other surgical wound in the body involving bone. In these wounds there exists all that is necessary for the regeneration of normal bone.

### NEW BONE FORMATION

There are three things necessary for the production of bone:

1. Blood vessels.
2. Building material.
    (a) A loose fibrous tissue.
    (b) A homeogenous (cartilage matrix), a granular or a necrotic material.
3. Stimulus, physiologic or pathologic.

There is an exceedingly rich blood supply in the cancellous bone of the jaws. Immediately after the operation of apicoectomy, the crypt is filled with blood which at once forms a clot and this becomes the building material or scaffolding through which new bone forms.

In uncovering these crypts after several months, we have found that the regenerated bone seems to be clinically identical with that immediately surrounding it. Our radiographic evidence in every case has corroborated our microscopical findings. In the radiograph may be seen the regular trabeculae and marrow spaces which are seen in normal bone which would clinically indicate that the new bone is similar in character to and receives its nutrition in the same manner as normal bone. It would seem that the final results obtained in bone regeneration after apicoectomy is comparable to the regeneration of bone following the extraction of a tooth.

The stimulus that is necessary for the regeneration of bone arises from the trauma produced by the operation. Opening into these regenerated areas six months, one year, two years and three years following the operation has convinced us from our bacteriological findings that they have remained sterile.—*Dental Items of Interest.*

